# Carboplatin combined with arsenic trioxide versus carboplatin combined with docetaxel treatment for LACC: A randomized, open-label, phase II clinical study

**DOI:** 10.1515/med-2024-1106

**Published:** 2024-12-12

**Authors:** Sijin Li, Yawen Zhang, Haiying Li, Zeyang He, An Li, Jiao Yan, Daocheng Li

**Affiliations:** Medical Department of Obstetrics and Gynecology, The First Affiliated Hospital of Guangzhou University of Chinese Medicine, Guangzhou, 510000, Guangdong, China; Gynecology, Baiyun Branch of the First Affiliated Hospital of Guangzhou University of Chinese Medicine, Guangzhou, Guangdong, China; Gynecology, Zhuhai Branch of Guangdong Provincial Hospital of Traditional Chinese Medicine, Zhuhai, Guangdong, China; Gynecology, Beijing University of Chinese Medicine Shenzhen Hospital (Longgang), Shenzhen, Guangdong, China; Respiratory Department, Shenzhen Hospital of Integrated Traditional Chinese and Western Medicine, Shenzhen, Guangdong, China

**Keywords:** locally advanced cervical cancer, arsenic trioxide, carboplatin, docetaxel, neoadjuvant chemotherapy

## Abstract

**Purpose:**

This study compared the efficacy and safety of carboplatin combined with arsenic trioxide versus carboplatin combined with docetaxel in treating locally advanced cervical cancer (LACC).

**Methods:**

A total of 48 patients were enrolled between January 2019 and December 2022 and randomly assigned to the experimental group (carboplatin + arsenic trioxide, *n* = 24) or control group (carboplatin + docetaxel, *n* = 24). The clinical efficacy, adverse reactions, and serological markers were analyzed.

**Results:**

There was no significant difference in baseline characteristics or total effective rates between the two groups (72.22% vs 68.42%, *P* > 0.05). Both groups showed significant reductions in serum squamous cell carcinoma antigen levels after chemotherapy (*P* < 0.05), but no significant difference was observed between groups (6.00 ± 11.36 ng/mL vs 8.42 ± 12.17 ng/mL, *P* > 0.05). Additionally, there was no significant difference in the incidence of adverse reactions (*P* > 0.05).

**Conclusion:**

Arsenic trioxide combined with carboplatin as a preoperative neoadjuvant chemotherapy for LACC is not worse than docetaxel combined with carboplatin in terms of short-term efficacy and safety during the treatment of LACC.

## 1 Introduction

Cervical cancer is the second most frequent common gynecological cancer in the world and is seriously harmful to the health of women with 500,000 new cases and hundred thousand deaths per year [1]. Locally advanced cervical cancer (LACC) accounts for about one third of all cervical cancer patients, while the disease is often diagnosed at an advanced stage. LACC is characterized by difficult surgery, easy recurrence, and metastasis. In spite of improvements in prevention and screening of LACC, approximately 67% of patients will have locally advanced stage and the 5-year survival rate is only 30–80% [2]. The spread of pelvic tumors to lymph nodes or distant metastasis is the main cause of poor prognosis in LACC. A significant number of patients will experience recurrence and die from metastatic disease. Using platinum-based regimen during concurrent chemoradiotherapy (CCRT) has been the standard of care for LACC according to the National Comprehensive Cancer Network (NCCN) guidelines [3]. Moreover, with a lack of radiotherapy equipment and severe radiotherapy-related complications, there is an increasing risk of occurrence of second tumor [4]. Some regions still prefer to recommend surgery after neoadjuvant chemotherapy (NACT), especially in young patients. Paclitaxel drugs combined with platinum drug regimens are commonly used in NACT. However, severe allergic reactions have occurred in some people taking paclitaxel drugs, and NACT is ineffective in certain cases leading to disease progression [5]. Therefore, it is necessary to find more effective NACT regimens for LACC patients. Traditional Chinese medicines arsenic trioxide (As_2_O_3_) have been used for hundreds of years, and recently it is widely accepted as an alternative treatment for acute promyelocytic leukemia (APL) and liver cancer [6]. It was found that degradation of retinoic acid receptor alpha (PML-RARα) oncogene was the main mechanism of As_2_O_3_ in the treatment of APL. At present, the anti-tumor mechanisms of As_2_O_3_ have been found to include but are not limited to, induction of caspase-3, generation of reactive oxygen species (ROS), alteration of the cell cycle, reduction of stem cell markers, and inhibition of glioma-associated oncogene family zinc finger (GLI) transcription factors [7]. However, whether it can be used in LACC patients is currently unknown. On this basis, our department started a phase II clinical study to explore the effect of As_2_O_3_ plus carboplatin in the therapy of LACC and compare the efficacy and safety of carboplatin combined with As_2_O_3_ versus carboplatin combined with docetaxel during neoadjuvant treatment.

## 2 Methods

### 2.1 Study design and participants

It was a single-center, phase II, open-label randomized prospective clinical trial (Clinical trial registration number: ChiCTR1900023822), and 44 LACC patients from the First Affiliated Hospital of Guangzhou University of Chinese medicine were enrolled to explore the effects of carboplatin combined with As_2_O_3_ versus carboplatin combined with docetaxel during neoadjuvant treatment. A central randomization system is used for experimental group (carboplatin combined with As_2_O_3_) and control group (carboplatin combined with docetaxel), respectively.

### 2.2 Study population

Patients with LACC were eligible for the trial. The inclusion and exclusion criteria have been set by the investigators.

Inclusion criteria included: (1) all patients need to sign an informed consent form; (2) aged 20–70 years old, female; (3) pathologically and radiographically confirmed as treatment-naive, LACC patients with stage IB2–IIB (the 2009 International Federation of Gynecology and Obstetrics [FIGO]) staging; (4) Karnofsky performance status scale ≥70; and (5) the blood cell counts, serum creatinine, aspartate aminotransferase, alanine aminotransferase, and bilirubin were normal.

### 2.3 Exclusion criteria

Patients who met any of the following criteria were excluded: (1) history of recurrence or distant metastasis; (2) history of surgery, chemotherapy, radiotherapy, or immunotherapy; (3) history of malignancies or concurrent malignancies; (4) females in pregnancy or lactation; (5) history of allergy to arsenic; and (6) previously participated in other clinical trials in the past 3 months.

### 2.4 Study endpoints

Study endpoints included: (1) deterioration; (2) severe bone marrow suppression; (3) history of surgery, chemotherapy, radiotherapy, or immunotherapy; (4) alanine transaminase or aspartate aminotransferase above three folds or creatinine above two folds, the upper limit of normal; (5) abnormal cardiac function during treatment, such as frequent ventricular premature beats; (6) patient who cannot tolerate the treatment, and side effects such as skin and mucosal lesions, where severe drug extravasation occurred; (7) metastasis during the treatment; and (8). poor compliance and cannot be followed up.

### 2.5 Randomization and masking

We randomly assigned eligible patients to either study group according to the ratio of 1:1. Therapy assignment was unmasked in the two following manners: (1) the investigators obtained informed consent from eligible patients and (2) the details of treatment allocations were opened sealed opaquely.

### 2.6 Procedures

All patients who were diagnosed underwent cervical biopsy. Pelvic examination was also performed to determine the stage of FIGO. Furthermore, blood tests, MRI, and ultrasonography of the abdomen and pelvis were also performed before random assignment. Patients received either two cycles of As_2_O_3_ (8 mg/m^2^) plus carboplatin once every 3 weeks or two cycles of docetaxel (75 mg/m^2^) plus carboplatin once every 3 weeks. The amount of carboplatin was calculated according to the region of curves 5–6. Clinical responses were evaluated after the second chemotherapy cycle. Patients who had no response or disease progression in the NACT received CCRT. However, if patients responded to the treatment of two cycle of NACT, they accepted Piver-Rutledge class III radical abdominal hysterectomy and underwent laparoscopic para-aortic lymph node dissection if necessary.

### 2.7 Statistical analysis

Statistical analysis was performed through the Statistic Package for Social Science (SPSS) 20.0 software (IBM, Armonk, NY, USA). The sample size of this study was small, and the data with multi-class attributes were used. Fisher’s exact test provides an accurate, reliable, and easy to implement statistical analysis method for small sample categorical data, which is especially suitable for pilot studies, exploratory studies, and studies conducted when large samples are not available. We therefore used Fisher’s test to analyze the data we obtained. Student’s *t*-test was carried out for the comparison of continuous variables by mean ± standard deviation, while *χ* ^2^ test was used to compare categorical variables. *P*-value < 0.05 was considered to be statistically significant.


**Ethical approval:** This study was approved by the Ethical Committee of The First Affiliated Hospital of Guangzhou University of Chinese Medicine (approval no. K-2019-023). This study was conducted in accordance with the Declaration of Helsinki and followed the ethical standards of China.
**Informed consent:** Signed written informed consents were obtained from the patients and/or guardians.

## 3 Results

### 3.1 Patient characteristics

A total of 48 patients with stage IB2-IIB cervical cancer were enrolled in this study between January 2019 and May 2022 and were randomly assigned to either the experimental group (carboplatin combined with As_2_O_3_, *n* = 24) or the control group (carboplatin combined with docetaxel, *n* = 24). Patient characteristics are summarized in Table 1. The data cutoff date for this analysis was May 31, 2022. All enrolled patients have signed the informed consent, and all relevant investigations were performed according to the principles of the Declaration of Helsinki. Eighteen patients among the experimental group had completed two courses of chemotherapy and surgical treatments, and five patients had completed two courses of chemotherapy (three of them were lost to follow-up and two patients requested transfer to another hospital for further treatment), and one patient was lost to follow-up after completing one course of chemotherapy. Meanwhile, 19 patients of the control group had completed two courses of chemotherapy and surgical treatments, three patients had completed two courses of chemotherapy (one patient was automatically transferred to a specialist hospital for treatment and two patients were lost to follow-up), and two patients had completed one course of chemotherapy (two patients were lost to follow-up) (Table 2).

**Table 1:** Baseline characteristics of the patients included in this study

	As_2_O_3_ plus carboplatin group (*n* = 24)	Docetaxel plus carboplatin group (*n* = 24)	*P*-value	*P*-value*
Age (years)	55.33 ± 8.96	54.83 ± 11.82	0.870	0.870
BMI (kg/m^2^)	23.70 ± 3.82	23.07 ± 3.90	0.560	0.560
Previous pregnancies	3.71 ± 1.58	3.88 ± 1.92	0.744	0.744
Previous live births	2.96 ± 1.55	3.00 ± 1.82	0.932	0.932
Previous miscarriages	0.75 ± 1.11	0.86 ± 0.95	0.884	0.884
Hypertension			0.7	1.000
Yes	6(25.00%)	5(20.83%)		
No	18(75.00%)	19(79.17%)		
Diabetes			0.551	1.000
Yes	2(8.33%)	1(4.17%)		
No	22(91.70%)	23(95.83%)		
Cardiopathy			0.296	0.609
Yes	3(12.50%)	1(4.17%)		
No	21(87.50%)	23(95.83%)		
Delivery method			0.532	1.000
Vaginal delivery	22(91.67%)	20(83.33%)		
Cesarean delivery	2(8.33%)	1(4.17%)		
FIGO stage			0.845	—
IB2	6(25.00%)	7(29.17%)		
IIA	12(50.00%)	10(41.66%)		
IIB	6(25.00%)	7(29.17%)		
Histological subtypes			0.600	—
Squamous cell carcinoma	22(91.67%)	23(95.83%)		
Adenocarcinoma	1(4.17%)	0(0.00%)		
Others**	1(4.17%)	1(4.17%)		

Data are mean ± SD/*n*(%). *P-values were calculated with independent *t*-test, if it is a continuous variable and calculated with unadjusted *χ* ^2^ test, if it is a categorical variable; **including adenosquamous carcinoma and clear cell carcinoma.

**Table 2:** Treatment accomplishment in both the groups

	As_2_O_3_ plus carboplatin group	Docetaxel plus carboplatin group
Two cycles of NACT plus surgery	18(75.00%)	19(79.17%)
Two cycles of NACT	5(20.83%)	3(12.50%)
One cycle of NACT	1(4.17%)	2(8.33%)

Data are *n*(%).

### 3.2 Tumor response

Response Evaluation Criteria in Solid Tumors, version 1.1CIST 1.1) was used to evaluate the clinical efficacy. This standard is the most widely accepted and used imaging response evaluation standard in clinical trials of solid tumors. The effect is divided into complete response, partial response, stable disease, or progressive disease [8]. The clinical effects are shown in Table 3. Compared to the control group, the total effective rates of the experimental group were 72.22 and 68.42%, respectively. There was no statistically significant difference between the two groups, *P* > 0.05.

**Table 3:** Short-term clinical efficacy in both groups

Treatment	*N*	Complete response + partial response	Stable disease		Progressive disease	Total effective rates (%)	*χ* ^2^	P*
As_2_O_3_ plus carboplatin	18	13(72.22%)	5(27.79%)		0	72.22	0.800	1.000
Docetaxel plus carboplatin	19	13(68.42%)	6(31.58%)		0	68.42		

Data are *n*(%). *P-values were calculated with Fisher’s exact test.

### 3.3 Serological response

Squamous cell carcinoma (SCC) is a protein over-expressed in the occurrence and development of cervical cancer, which is often used to evaluate the prognosis of cervical cancer [9]. The level of SCC was decreased significantly before and after chemotherapy in both the groups. Compared with before chemotherapy, there was a statistical difference in the decrease of SCC after chemotherapy, *P* < 0.05 (Tables 4 and 5). While comparing the decline of SCC before and after treatment in the two groups, there was no statistical difference between the two groups, *P* > 0.05 (Table 6).

**Table 4:** Comparison of serum SCC before chemotherapy and before surgery in As_2_O_3_ plus carboplatin group

SCC (ng/mL)	*N*	Before chemotherapy	Before surgery	*t*	*P*
Normal	5	1.02 ± 0.40	1.08 ± 0.35	−0.548	0.806
Abnormal	13	18.62 ± 20.65	12.62 ± 15.84	0.831	0.414

Data are mean ± SD/*n*(%).

**Table 5:** Comparison of serum SCC before chemotherapy and before surgery in docetaxel plus carboplatin group

SCC (ng/mL)	*N*	Before chemotherapy	Before surgery	*t*	*P*
Normal	8	0.91 ± 0.33	0.79 ± 0.43	0.655	0.655
Abnormal	11	13.37 ± 20.28	4.95 ± 9.47	2.295	0.045

Data are mean ± SD/*n*(%).

**Table 6:** Serum SCC in both the groups

SCC (ng/mL)	As_2_O_3_ plus carboplatin group	Docetaxel plus carboplatin group	*t*	*P*
Before chemotherapy	18.62 ± 20.65	13.37 ± 20.28	0.626	0.538
Before surgery	12.62 ± 15.84	4.95 ± 9.47	1.406	0.174
Difference value	6.00 ± 11.36	8.42 ± 12.17	−0.505	0.619

Data are mean ± SD/*n*(%).

### 3.4 Intraoperative bleeding and duration of operation

The intraoperative bleeding and duration of operation of the two groups were contrasted. The average intraoperative bleeding of the experimental group and the control group was 547.22 ± 475.74 mL vs 533.16 ± 486.65 mL, *P* > 0.05), and the average duration of operation was 224.67 ± 32.87 min vs 225.32 ± 64.11 min, *P* > 0.05, there were no statistically significant difference between the two groups (Table 7).

**Table 7:** Surgery related indicators in both the groups

	As_2_O_3_ plus carboplatin group	Docetaxel plus carboplatin group	*t*	*P*
*N*	18	19		
Intraoperative bleeding (mL)	547.22 ± 475.74	533.16 ± 486.65	0.09	0.929
Length of surgery (min)	224.67 ± 32.87	225.32 ± 64.11	0.038	0.970

Data are mean ± SD/*n*(%).

### 3.5 Safety and toxicity

Hematological indicators before the first chemotherapy were used as baseline data in patients who have completed two chemotherapy sessions and surgery, and the hematological indicators before the second chemotherapy and surgery served as indicators of hematological changes under the action of chemotherapy drugs. While for the patients who have completed only two chemotherapy sessions, hematological indicators before the first chemotherapy were used as baseline data, and hematological indicator changes before and after the second chemotherapy were collected. For patients who have completed only one chemotherapy session, the hematological indicators before the first chemotherapy were used as baseline data, and the changes of hematological indicators after the first chemotherapy were collected. In summary, a total of 84 hematology evaluation results were collected for statistical analysis, as shown in Table 8. For the incidence of adverse reactions in the blood system and liver and kidney function, the comparison between the two groups of patients was also not statistically different (*P* > 0.05). The gastrointestinal adverse reactions that occurred during the two chemotherapy treatments in the two groups were also collected and there were no significant difference between the two groups (Table 9). Others adverse reactions that occurred during the two chemotherapy treatments in the two groups were also collected and there was no visible statistical difference between the two groups (Table 10).

**Table 8:** Hematological adverse events reported during treatment in both the groups

	As_2_O_3_ plus carboplatin group	Docetaxel plus carboplatin group	*P*-value	*P*-value*
*N*	47	45		
Leucopenia			0.114	
0	33(70.21%)	28(62.22%)		
I	12(25.53%)	8(17.77%)		
II	2(4.26%)	7(15.55%)		
III	0(0.00%)	2(4.44%)		
Neutropenia			0.360	
0	31(65.96%)	23(51.11%)		
I	10(21.28%)	12(26.67%)		
II	5(10.64%)	6(13.33%)		
III	1(2.13%)	4(8.88%)		
Anemia			0.137	
0	29(61.70%)	34(75.56%)		
I	15(31.91%)	11(24.44%)		
II	3(6.38%)	0(0.00%)		
Thrombocytopenia			0.325	1.000
0	46(97.87%)	45(100.00%)		
I	1(2.13%)	0(0.00%)		
Hepatotoxicity			0.577	
0	42(89.36%)	42(93.33%)		
I	4(8.51%)	3(6.66%)		
II	1(2.13%)	0(0.00%)		
Nephrotoxicity			0.184	0.362
0	43(91.49%)	44(97.77%)		
I	4(8.51%)	1(2.22%)		

Data are *n*(%). *P-values were calculated with Fisher’s exact test.

**Table 9:** Gastrointestinal adverse events reported during treatment in both the groups

	As_2_O_3_ plus carboplatin group	Docetaxel plus carboplatin group	*P*-value	*P*-value*
*N*	47	45		
Nausea				0.738
0	26(55.32)	29(62.22%)		
I	3(6.38%)	4(8.88%)		
II	14(29.79%)	9(20.00%)		
III	4(8.51%)	4(8.88%)		
Diarrhea			0.325	1.000
0	46(97.87%)	45(100.00%)		
I	1(2.13%)	0(0.00%)		
Constipate			0.450	0.575
0	38(80.85%)	39(86.67%)		
I	9(19.15%)	6(13.33%)		

Data are *n*(%). *P-values were calculated with Fisher’s exact test.

**Table 10:** Others adverse events reported during treatment in both the groups

	As_2_O_3_ plus carboplatin group	Docetaxel plus carboplatin group	*P*-value	*P*-value*
*N*	47	45		
Allergic reaction			0.975	1.000
0	46(97.87%)	44(97.88%)		
I	1(2.13%)	1(2.22%)		
Ache			0.300	
0	41(87.23%)	39(86.67%)		
I	6(12.77%)	4(8.88%)		
II	0(0.00%)	2(4.44%)		
Mucositis			0.325	1.000
0	46(97.87%)	45(100.00%)		
I	1(2.13%)	0(0.00%)		
Neurotoxicity			0.162	0.495
0	45(95.74%)	45(100.00%)		
I	2(4.26%)	0(0.00%)		

Data are *n*(%). *P-values were calculated with Fisher’s exact test.

## 4 Discussion

Our innovative use of As_2_O_3_ combined with carboplatin in the treatment of LACC has produced similar efficacy and tolerable side effects as docetaxel combined with carboplatin, which provides a new insight for cervical cancer’s treatment. This study aims to evaluate the clinical effects of As_2_O_3_ combined with carboplatin in LACC and compared with paclitaxel combined with carboplatin drugs to explore another effective, safe, and economical drug for LACC. Our data showed that there was no significant difference in the efficacy and safety between the two groups. The total effective rates of the experimental group and the control group were 72.22 and 68.42%, respectively. The serum SCC was also not statistically significant between the two groups. In addition, the average blood loss during operation, the average operation time, and the incidence of adverse reactions were also not statistically different. These data indicated that As_2_O_3_ has been shown to successfully improve survival outcomes in patients with LACC. Although our data provided another option for the treatment of LACC, there are still some limitations in the research process. The number of patients included in this study is limited, and more samples are needed to further prove the effectiveness and universality of this therapy. Unfortunately, due to the dropout of patients in this study, we did not obtain enough information from this study. In addition, this study only collected the adverse reactions of chemotherapy in patients during hospitalization. Due to the large difference in hospitalization time between the two groups, the 18 patients in the experimental group had much longer hospital stay than those in the control group, so the safety evaluation may be biased. Therefore, we expect to further improve the study of long-term side effects of chemotherapy during follow-up. Our data show that the As_2_O_3_ plus carboplatin provides more options for the treatment of LACC.

As_2_O_3_ is one of the effective components of arsenic in traditional Chinese medicine. Ben cao gang mu (Compendium of Materia Medica) has recorded that “arsenic is a poisonous medicine.” This is because As_2_O_3_ has a narrow therapeutic window, and it is easy to produce toxic side effects. But with the right preparation method and appropriate dosage, As_2_O_3_ can be used to treat tumors as a traditional Chinese medicine. Since the 1970s, clinical studies in China showed that As_2_O_3_ is an effective treatment for APL patients in obtaining high clinical complete remission rates and small side effects such as bone marrow suppression. In 2001, it was approved by the US Food and Drug Administration for the treatment of APL [6]. Later studies found that As_2_O_3_ has been extended for the treatment of breast cancer [10], lung cancer [11,12], gastric cancer [13,14], liver cancer [15,16], bladder cancer [17], cervical cancer [18], and other solid tumors. As_2_O_3_ has also been demonstrated to induce significant clinical benefits of antitumor effects in a wide range of solid tumors including gallbladder cancer [19], pancreatic cancer [20], lung cancer [21], and liver cancer. Due to the lower cost and smaller side effects, As_2_O_3_ has attracted more and more attention in the gynecological tumor. Currently research studies have demonstrated the role of As_2_O_3_ in suppressing the proliferation of tumor cells and induce mitochondria apoptosis [22,23] or up-regulating intracellular ROS induced lipid peroxidation [24] of cervical cancer cells both *in vitro* and *in vivo*. The NCCN guideline insights show that the combination with platinum-based chemotherapy drugs serve as effective drugs for the treatment of cervical cancer [25]. The combination of carboplatin and docetaxel is an effective and well-tolerated treatment for patients with advanced cervical cancer. However, whether the combination of As_2_O_3_ and carboplatin has the same anti-tumor effect and less side effects compared to the combination of carboplatin and docetaxel needs further clinical study. Previous clinical trials have demonstrated that the synergism of As_2_O_3_ and platinum drugs inhibited the cell growth and induced cell apoptosis of tumor cells in a dose-dependent manner, such as oral cancer cells [26], hepatocarcinoma cells [27], colorectal cancer cells [28], and non-small cell lung cancer cells [29]. *In vivo* experiments also show that As_2_O_3_ combined with platinum drugs can inhibit the growth of transplanted tumors in mouse model [30]. Moreover, there is no increased toxic side effect in this combination [31]. Based on the experience of As_2_O_3_ combination both *in vitro* and *in vivo* of tumor model, we innovatively used As_2_O_3_ combined with carboplatin to treat LACC patients, and observed its efficacy and safety.

Conclusively, we found that As_2_O_3_ combined with carboplatin, as a chemotherapy regimen for LACC is not worse than docetaxel combined with carboplatin in terms of short-term efficacy and safety during the treatment of LACC. It provides a new chemotherapy regimen for the treatment of LACC patients, and also provides a new idea for the treatment of advanced and recurrent cervical cancer.

## Abbreviations

APL: acute promyelocytic leukemia
CCRT: concurrent chemoradiotherapy
FIGO: Federation of Gynecology and Obstetrics
LACC: locally advanced cervical cancer
NACT: neoadjuvant chemotherapy
NCCN: National Comprehensive Cancer Network
